# Supplementary material for: Acceptance and compliance with micronutrient powder (MNP) among children aged 6–23 months in northern Nigeria
Source: PLOS Glob Public Health. 2022 Oct 17;2(10):e0000961. doi: 10.1371/journal.pgph.0000961 (PMC10022258; doi:10.1371/journal.pgph.0000961)
Supplement: S1 Dataset — (ZIP) [file pgph.0000961.s008.zip › S8_Dataset/Spot Checks.zip!/Spot Checks/09072015_A_GA_PM_2_SC.pdf]

SPOT CHECK RECORDING FORM

State: _ADAMAWA_______________ LGA: ___GANYE______________ Community: ___SUGU__________________
NAME OF CAREGIVER…………MAGRET MOSES

Sex of index child: _F_____________ Age of index child (in months): __17________________

Spot Check Information

1. **Date of MNP distribution to household** (circle option below based on LGA name above)**:**
   - **Kebbi:** BirninKebbi&Jega LGA: **June 16**
   - **Kebbi:** Danko-wasagu: **June 17**
   - **Adamawa:**Song, Ganye: **June 18**
   - **Adamawa:**Guyuk **June 19**
2. **Date of Spot Check Observation: __09-07-2015_________________**
3. **Number of sachets remaining**(Ask the caregiver to show you and count the specific number of sachets remaining)**:**

   53

4. **Number of sachets that should remainusing one MNP sachet per day** (Analyst only)**:**

   40

5. **Number of sachets remaining / number of sachets should be remaining**(Analyst only)**:**

   ___53______ / __40_______ = _____132.5____ %

Probes

Question 1. You have **XX** MNP sachets remaining. Explain how easy or difficult it was to use the MNP product on a daily basis for your child 6 – 23 months? (Circle one answer)

Very Difficult **Difficult** **Easy** **Very Easy**

Question 2. Describe any specific challenges that you faced that made the MNP difficult to use on a daily basis?THIS MY CHILD DOES NOT LIKE KUNU AT ALL…..SO I FIND IT DIFFICULT TO GIVE HER THE POWDER……..SOMETIMES I HAVE TO FORCE HER…….I GUESS SHE DOES NOT LIKE THE TASTE OF THE POWDER.

Question 3. Describe specific aspects of the MNP product that you or your child enjoyed that made it easier to use on a daily basis? FOR NOW THER IS NONE,THAT IS WHY YOU SHOULD HAVE NOTICE THAT THE MNP POWDER IS MUCH BECAUSE SHE IS REFUSING TO TAKE IT

Question 4. What questions do you have about the MNP based on your experience using it? **APART FROM KUNU,CAN THIS MNP BE USED WITH ANY OTHER MEAL?**

Question 5. What specific recommendations do you have that we can do as a program to make the MNP easier to use on a daily basis? THEY SHOULD MAKE THE MNP VERY SWEET IN TASTE……..IF POSSIBLE,THE MNP SHOULD BE IN FORM OF A SWEET(CANDY),SO THAT THE CHILD CAN LICK IT INSTEAD OF MIXING IT IN A FOOD.
